# Religio Medici

**Published:** 1887-08-20

**Authors:** 


*(To be continued.)*

### RELIGIO MEDICI.

*(Continued from p. 328, vol. ii.)*

ALL Browne's lapses from orthodoxy are on the humanitarian side. The heresies of mere enthusiasm do not lead him astray, nor has he any tendency to drift into the arid paths of materialism. "As for those wingy mysteries of divinity and airy subtleties in religion which have unhinged the brains of better heads, they never stretched the *pia mater* of mine." Yet he likes to contemplate the supernatural, and for a sufficiently quaint reason : "I desire to exercise my faith in the difficultest point ; for to credit ordinary and visible objects is not faith, but persuasion. I would not have been one of those Israelites that passed the Red Sea, nor one of Christ's patients upon whom He wrought His wonders ; then had my faith been thrust upon me ; nor should I enjoy that greater blessing pronounced to all that believe and saw not." He admits that doubts may arise, but says: "I can answer all the objections of Satan and my rebellious reason with that odd resolution I learned of Tertullian—*Certum est quia impossibile est.*" But this calm certainty has not been easily attained. "There are, as in philosophy, so in divinity, sturdy doubts and boisterous objections, wherewith the unhappiness of our knowledge too nearly acquainteth us. More of these no man hath known than myself; which I confess I conquered, not in a martial posture, but on my knees."

So strenuous and eager is the faith so gained that it demands even what we should consider unnecessary food. Sir Thomas is not content to accept an event as miraculous when no other explanation will suffice ; he likes to make a miracle where the ordinary course of nature demands none. "The devil," he says, "takes a hint of infidelity from our studies," and gives natural causes for apparently supernatural effects. For example,—"Having seen some experiments of bitumen, and having read far more of naphtha, he whispered to my curiosity the fire of the altar might be natural, and bade me mistrust a miracle in Elias, when he intrenched the altar round with water, for that inflammable substance yields not easily unto water, but flames in the arms of its antagonist. And thus would he inveigle my belief to think the combustion of Sodom might be natural, and that there was an asphaltic and bituminous nature in that lake before the fire of Gomorrah." But our author resists the temptation to accept the natural solution of these events, and clings to his miracle. It was the best proof of faith one of his time could give. The evolution of the human soul had not then gone so far that it could realise the miracles of daily life. It was not given to the seventeenth century to perceive that the laws of God's providence are more wonderful than the apparent exceptions. Portents and unforeseen accidents were supposed to be the highest displays of His power. The closer and not less reverent study of His works, which teaches us to marvel as much "at a rose's birth, as at a comet's rush," was not for the men of those days.

In his days of greatest doubt, however, Sir Thomas protests that he was never inclined to any "desperate positions of atheism." Indeed, he will not admit that such a thing as atheism really exists. His mind has an insight beyond the common perceptions of his time, and sees the truth underlying the formulæ of different creeds. "That doctrine of Epicurus, that denied the providence of God, was no atheism," he protests ; "but a magnificent and high-strained conceit of His majesty, which he deemed too sublime to mind the trivial actions of those inferior creatures. That fatal necessity of the Stoics is nothing but the immutable law of His will."

In short, his wise charity refuses to trouble itself with those fine and unimportant questions which trouble fretful and over-sensitive souls. In all the heretical books he has read he has found nothing which, if rightly understood, need startle "a discreet belief." He grasps the essential, and lets the rest go by. As he says, with a touch of humour, "I can read that Lazarus was raised from the dead, yet not demand where, in the interim, his soul awaited ; or raise a law-case, whether his heir might lawfully detain his inheritance bequeathed unto him by his death ; and he, though restored to life, have no plea or title to his former possessions." He perceives the unimportance of what may be termed material theology, facts or fancies having no bearing on man's conduct, and conveying no guidance to his soul, and calmly ignores it. His dictum on that subject deserves to be kept in remembrance : "There are a bundle of curiosities, not only in philosophy but in divinity, proposed and discussed by men of most supposed abilities, which indeed are not worthy our vacant hours, much less our serious studies."

Consequently, books filled with frivolous criticism and proofs of irrelevant propositions find small favour in the doctor's eyes. Indeed, he inclines to think there were already in his day too many books in the world, though he added several to the number. What would he say in ours ? "Of those three great inventions in Germany," he remarks, alluding to gunpowder, printing, and the mariner's compass, "there are two which are not without their incommodities"; and he longs to "condemn to the fire those swarms and millions of rhapsodies, begotten only to distract and abuse the weaker judgments of scholars, and to maintain the trade and mystery of typographers."

Without doubt Sir Thomas would readily have burned many modern books which consist only of amplifications of ideas he himself enunciates. Passing by that allusion to the æsthetic damsel of a pre-æsthetic century who lived "without meat, on the smell of a rose," we may well think over his serious explanation of the oft-repeated idea that suicide is cowardly. "It is a brave act of valour to contemn death ; but, where life is more terrible than death, it is then the truest valour to live : and herein religion hath taught us a noble example ; for all the valiant acts of Curtius, Scævola, or Codrus, do not parallel or match that one of Job ; and sure there is no torture to the rack of a disease, nor any poniards in death itself, like those in the way of prologue into it."

The doctor respects life, as those do who have made a study of its phenomena ; but the thought of death is not terrible to him, but sweet and restful. "Men that look no further than their outsides," he says, "think health an appurtenance unto life, and quarrel with their constitutions for being sick ; but I, that have examined the parts of man, and know upon what tender filaments that fabric hangs, do wonder that we are not always so ; and considering the thousand doors that lead to death, do thank my God that we can die but once. It is in the power of every hand to destroy us, and we are beholden unto every one we meet he doth not kill us. There is, therefore, but one comfort left, that though it be in the power of the weakest to take away life, it is not in the strongest to deprive us of death."

Among other ideas which we consider modern, Browne had tested that of "following goodness for good's sake rather than for God's," and found it wanting. "I have tried if I could reach that great resolution to be honest without a thought of heaven or hell ; and indeed I found upon a natural inclination, and inbred loyalty unto virtue, that I could serve her without a livery, yet not in that resolved and venerable way, but that the frailty of my nature upon an easy temptation might be induced to forget her." Therefore he holds to the idea of rewards and punishments, but in no material sense. He believes in no fixed and geographical hell. "Men speak too popularly," he declares, "who place it in those flaming mountains, which to grosser apprehensions represent hell. The heart of man is the place the devils dwell in. I feel sometimes a hell within myself. Lucifer keeps his court in my breast. Legion is revived in me. A distracted conscience here is a shadow or introduction to hell hereafter."

But he does not dwell much on the darker side of religion. His soul seeks guidance from above, not warning from below. "I have so fixed my contemplations on heaven," he tells us, "that I have almost forgot the idea of hell, and am afraid rather to lose the joys of the one than endure the misery of the other. To be deprived of them is a perfect hell, and needs, methinks, no addition to complete our afflictions. That terrible term hath never detained me from sin, nor do I owe any good action to the name thereof. I can hardly think there was ever any scared into heaven : they go the fairest way to heaven that would serve God without a hell. Other mercenaries, that crouch unto Him in fear of hell, though they term themselves the servants, are indeed but the slaves of the Almighty."

*(To be continued.)*

### THE STORY OF THE HOSPITALS.

*(Continued from p. 276, vol. ii.)*

